# Reporting Emerging Resistance of *Streptococcus pneumoniae* from India

**DOI:** 10.4103/0974-777X.59245

**Published:** 2010

**Authors:** Kiran Chawla, Bimala Gurung, Chiranjay Mukhopadhyay, Indira Bairy

**Affiliations:** *Department of Microbiology, Kasturba Medical College, Manipal, Karnataka, India*

**Keywords:** Minimum inhibitory concentration, Multi-drug resistant strains, Penicillin, Resistance, *Streptococcus pneumoniae*

## Abstract

**Background:**

There are reports of emergence of resistant strains of *S. pneumoniae* showing resistance to penicillin from all over the world, and now, resistance to multiple drugs (multidrug-resistant strains) has been added to it. However, scanty reports are available so far from India, depicting such resistance.

**Aims:**

The aim of the present study is to look for the prevalence of penicillin-resistant pneumococci and also the multidrug-resistant strains among *S. pneumoniae*, isolated from respiratory specimens, in the coastal part of South India.

**Settings and Design:**

A cross-sectional study was conducted from June 2008 to December 2008, in our tertiary care center. Fifty pathogenic clinical isolates were collected from patients suffering from lower respiratory tract infections.

**Materials and Methods:**

Penicillin resistance was screened by 1 μg oxacillin disk on Muller-Hinton blood agar followed by Minimum Inhibitory Concentration (MIC) detection by the agar dilution method according to the Clinical Laboratory Standards Institute (CLSI) guidelines. Antibiotic susceptibility for other antibiotics was carried out by the Kirby Bauer disk diffusion method followed by an E-test with HiComb test strips from Hi-media.

**Results:**

Out of 50 isolates, 4% (95% Confidence Interval - 1.4, 9.4) showed total resistance to penicillin, whereas, 10% (95% CI; 1.6, 18.3) showed intermediate resistance. These penicillin-resistant pneumococci (4%) were also found to be multidrug-resistant (MDR) strains. Maximum resistance was observed for cotrimoxazole and tetracycline (24% each with 95% CI; 12.2, 35.8) followed by erythromycin and ciprofloxacin (14% each with 95%CI; 4.4, 23.6).

**Conclusions:**

Increasing emergence of the resistant strains of *S. pneumoniae* in the community set up requires continuous monitoring and a restricted use of antibiotics to keep a check on its resistance pattern, for an effective treatment plan.

## INTRODUCTION

*Streptococcus pneumoniae* is the most common cause of community-acquired respiratory tract infections such as otitis media, sinusitis, and pneumonias.[1] Globally, pneumococcal diseases account for 1 to 2 million deaths annually in both extremes of age.[2] It is supposed to be a very sensitive organism to routine antibiotics especially to penicillins. However, with the isolation of the first clinically significant penicillin-resistant pneumococcus (PRP) in 1967, many studies from different parts of the world have reported an increasing emergence of PRP.[3] At present there are not only reports of resistant strains of *S. pneumoniae* to the beta lactam group of antibiotics, but there is also an emergence of multidrug-resistant strains.[4]

Of late, in a US study, for isolates collected between 2000 and 2004, 21.2% resistance for penicillin has been reported.[5] Another recent survey of eight European countries has observed penicillin resistance as 24.6% in *S. pneumoniae*,[6] whereas, a study done in Australia[3] revealed 6.7% penicillin resistance. A Malaysian study has depicted 21.6% penicillin resistance with 30% strains showing penicillin intermediate sensitivity.[7] Increasing emergence of resistant strains of *S. pneumoniae* is of major concern, especially in cases of meningitis, as it can lead to treatment failures; moreover, it prolongs the stay in the hospital, thus increasing the morbidity and mortality. PRP contain low-affinity penicillin binding proteins and also often produce abnormal indirectly cross-linked cell walls.[8] In India there are only few reports that show the resistance pattern in *S. pneumoniae*. Surveillance for resistance of *S. pneumoniae* has noticed the upsurgence of intermediate sensitivity from CMC Vellore in the southern part of India,[9] whereas, a study done in North India[10] has shown 2.3% resistance. Another study from South India has reported low-level resistance, although they could not find out any strain showing absolute resistance.[11] Yet another collaborative study from eight Asian countries including India has revealed 35.1% total resistance in *S. pneumoniae*.[12] Hence, this study has been done with the objective of finding out the prevalence of PRP strains giving rise to respiratory infections in the region of coastal Karnataka, India. This study has also been aimed at finding out multidrug resistance strains among the isolated PRP, so as to formulate the treatment plan for the infection caused by this bacterium.

## MATERIALS AND METHODS

The present cross-sectional study was done from June, 2008 to December, 2008 in a tertiary care teaching institution in coastal Karnataka, India.

### Collection of bacterial isolates

Fifty clinical isolates of *S. pneumoniae* were isolated from respiratory samples (sputum and bronchoalveolar lavages) collected from adult in- patients suffering from community-acquired lower respiratory infection. The sputum samples that satisfied the Bartlett grading were selected for study. The organism was identified with gram staining, hemolysis on sheep blood agar, bile solubility, and sensitivity to optochin. Demographic and clinical details such as age, sex, type of infection, underlying disease, and response to treatment, for all 50 patients were noted down and evaluated.

### Antibacterial susceptibility testing by disk diffusion method

The antibiotic susceptibility testing was done first by using the Kirby-Bauer disk diffusion method on Muller-Hinton sheep blood agar (MH-SBA). Screening for penicillin resistance was done by using the oxacillin (1 μg) disk. Other antimicrobials that were tested include tetracycline (30 μg), erythromycin (15 μg), ciprofloxacin (5 μg), cotrimoxazole (25 μg), and cefotaxime (30 μg) according to the Clinical Laboratory Standard Institute (CLSI) guidelines.[13]

### Determination of minimum inhibitory concentration

Minimum Inhibitory Concentration (MIC) for penicillin was done using the agar dilution method for strains showing resistance to 1 μg oxacillin. MIC for all other above-mentioned antibiotics was determined by the E-test, using HiComb test strips (Hi media, India). The strains were divided into resistant, intermediate or sensitive according to the CLSI guidelines. *S. pneumoniae* strains showing resistance to penicillin along with two or more non beta-lactam antibiotics were labeled as multi-drug resistant strains (MDR).[4]

### Statistical analysis

Statistical analysis was done using the software SPSS (Statistical Package for Social Sciences) version 15.0 for windows. Proportions have been calculated for categorical variables and data has been presented in the form of 95% Confidence Interval (CI). Chi-square test and *t*-test were used to study difference in proportions and means, respectively.

## RESULTS

### Demographic and clinical details

The patients were in the age range of 15-79 years, with 56% of the patients more than 50 years of age and 34% between 30 and 50 years. Male to female ratio was 2.8:1. Clinically 20 patients (40%) were suffering from chronic obstructive pulmonary disease, 23 (46%) from community-acquired pneumonia, 4 (8%) from bronchiectasis, and 3 (6%) from aspiration pneumonia. Table 1 shows the difference in the demographic and clinical profiles of patients having infection with penicillin-sensitive and resistant isolates of *S. pneumoniae*. Patients having infection with resistant isolates were having either neoplastic or chronic kidney disease as the underlying illness.

**Table 1:** Demographic and clinical profile of patients with *Streptococcus pneumoniae* infection

	Patient with sensitive isolates no. = 43	Patients with resistant isolates no. = 7	*P* value
Males n (%)	32/37 (86.5)	5/37 (13.5)	0.786
Age (in years)	50.4 (16.01)	50.6 (21.2)	0.983
Mean (SD)			
Chronic underlying disease n (%)			
Bronchopulmonary disease	31 (91.2)	3 (8.8)	0.032
Neoplastic disease	4 (66.7)	2 (33.3)	
Liver disease	1 (100)	0	
Kidney disease	0	1 (100)	
Duration of hospital stay in days	5.41 (3.16) (n = 17)	5 (1.41) (n = 4)	0.805
Mean (SD)			

SD = Standard deviation

### Disk diffusion method

Maximum resistance was shown for cotrimoxazole (36%) followed by tetracycline (38%), cefotaxime (30%), penicillins (14%), ciprofloxacin (14%) and erythromycin (14%). Intermediate resistance for cotrimoxazole was observed in 20% strains, followed by ciprofloxacin, tetracycline and erythromycin in 16,14 and 6%, respectively.

### Minimum inhibitory concentration

Minimum inhibitory concentration (MIC) by the agar dilution method revealed 4% (95% CI; -1.4,9.4) resistance for penicillin with 10% strains (95% CI; 1.6,18.3) showing intermediate sensitivity. Maximum resistance was observed for cotrimoxazole and tetracycline (24% each with 95% CI; 12.2,35.8) and least for cefotaxime (0%). Intermediate resistance was seen in another 24% of the strains for cotrimoxazole. Resistance for erythromycin and ciprofloxacin was seen in 14% of the strains each (95% CI; 4.4,23.6). MDR was noticed in two (4%) strains of *S. pneumoniae*. Twelve (24%) strains showed resistance to only one antibiotic. Table 2 depicts the comparison of resistance shown by *S. pneumoniae* in the present study with the studies done in the past. Table 3 shows the MIC for other drugs, for isolates that showed total and intermediate resistance to penicillin.

**Table 2:** Comparison of resistance shown by *S. pneumoniae* in the present study with earlier studies

Country*/Year	E (%)	Pn (%)	Ct (%)	Cf (%)	Co-tri (%)	T (%)
Present study	14	4 R 10 IM	0	14	24	24
South India^9^ (1995)	-	4.6	-	-	-	-
South India^11^ (1996-2000)	4.6	7.3IM	-	-	36	12.6
North India^10^(1999-2002)	-	18.3	1.7	-	61.7	76.7
Malaysia^7^ (1999-2003)	39.2	21.6	17.5	-	-	-
Eight Asian countries^12^ (2002-2004)	56.1	35.1	7**	0***	-	-
Australia^3^ (1994-95)	11	6.7	-	-	42	15
USA^5^ (2000-04)	29.3	21.2	24.07	0.9	28.25	15.05
UAE^29^(2004-06)	30	5	-	24****	77	16.9

Cf = Ciprofloxacin; Co-tri = Cotrimoxazole; Ct = Cefotaxime; E = Erythromycin; Pn = Penicillin; T = Tetracycline; IM = Intermediate resistant; R = Resistant;

* =Reference number;

** =checked for ceftriaxone;

*** =checked for levofloxacin;

**** =checked for ofloxacin

**Table 3:** Penicillin-resistant and intermediate-resistant isolates of *S. pneumoniae* showing MIC (mg/L) for other antibiotics

Pn	E	Ct	Cf	Co-tri	T
2 (R)	>4 (R)	0.1 (S)	0.25 (S)	>4 (R)	0.01 (S)
1 (IM)	4 (R)	0.1 (S)	0.25 (S)	2 (IM)	0.1 (S)
2 (R)	0.1 (S)	0.1 (S)	10 (R)	240 (R)	30 (R)
1 (IM)	0.1 (S)	0.1 (S)	0.25 (S)	>4 (R)	3 (IM)
1 (IM)	4 (R)	1 (S)	5 (IM)	>4 (R)	10 (R)
1 (IM)	1 (S)	1 (S)	5 (IM)	4 (R)	240 (R)
1 (IM)	1 (S)	0.1 (S)	30 (R)	60 (R)	0.01 (S)

Cf = Ciprofloxacin; R = Resistant; Co-tri = Cotrimoxazole; IM = Intermediate resistant; Ct = Cefotaxime; S = Sensitive; E = Erythromycin; Pn = Penicillin; T = Tetracycline

### Antimicrobial therapy

The information on antimicrobial therapy was available for 36 patients. In 28/36 patients (77.7%) monotherapy was given as an empirical treatment. Of these, penicillin (23/28, 82.1%) was the most commonly prescribed antibiotic followed by quinolone and third generation cephalosporin (2/28, 7.1% each) followed by doxycycline (1/28, 3.6%). Combination therapy was prescribed in 8/36 patients (22.2%). Out of these, penicillin + aminoglycoside + quinolone was given in 75% (6/8) cases followed by third generation cephalosporin + aminoglycoside in 25% (2/8) cases.

## DISCUSSION

Higher rate of isolation of *S. pneumoniae*, from patients above 56 years of age, suffering from lower respiratory infections corresponds with the existing fact that the bacterium is common in people with reduced immunity and in extremes of age.[14] Underlying chronic illnesses in turn further lower the immune status and favor the growth of the pathogen.

Penicillin resistance among *S. pneumoniae* is a global problem. Laboratory mutants of pneumococci resistant to penicillin were selected as early as the 1940s.[15,16] It was 20 years before the first clinical isolate, with reduced susceptibility to penicillin, was reported from Boston, Massachusetts.[17] A 4% total resistance to penicillin and 10% intermediate resistance, as observed in the present study, shows the increasing emergence of resistance strains of *S. pneumoniae* in India. Earlier, a three-year surveillance for penicillin resistance from Vellore revealed 4.6% of intermediate resistance to penicillin,[9] whereas, a North Indian study reported 15.2% (26/170) intermediate resistance and 2.3% (4/170) penicillin resistance.[10] The difference in the resistance pattern of *S. pneumoniae* as observed in South and North Indian studies has been explained by Lalitha *et al*. on the basis of the high genetic diversity that exists among strains isolated from different geographical areas within India.[18]

Screening with oxacillin (1 μg) disks was found adequate to pick up strains of *S. pneumoniae* with reduced susceptibility to penicillin, 10 years back.[19] However, it has been found later that it cannot distinguish between intermediate resistance strains and borderline susceptible strains.[20,21] It was therefore recommended that MIC determination by the agar or the broth dilution method should be performed for strains showing resistance to oxacillin using the disk diffusion technique. This might be the reason why we observed penicillin resistance by agar dilution in two strains (MIC 2 μg/ml), in contrast to seven strains (14%), by using disk diffusion testing. Although total resistance for penicillin is less in India as compared to other countries, increase in the number of intermediate resistant strains in the present study is also a matter of concern, as it can result in a greater spread of resistance strains in the near future. These strains can lead to a higher rate of treatment failures, thereby, increasing the hospital stay, especially in cases of meningitis. Moreover, it is also known that strains showing penicillin resistance can have genes responsible for resistance to other antibiotics.[10] It is rightly suggested, therefore, that the inadvertent and injudicious use of antibiotics can be a cause for upcoming resistance, as documented.[22]

The first MDR strain was isolated from the purulent sputum of a child who, in July 1977, at Baragwanath Hospital, Johannesburg, developed pneumonia following a previous therapy with penicillin and cephalothin.[23,24] High-level penicillin resistance and multiple resistance was first recognized only among serotype 6A and 19A pneumococci strains in South Africa.[25] At present, MDR strains belong predominantly to the serogroup/type 6, 9, 14, 23 as seen in the western world.[26,27] Prolonged carriage and rapid reacquisition provide an increased chance of exposure to antibiotics and thus may be important selective factors in predisposing these particular serogroups to antibiotic resistance, in hospital settings. The present study has observed MDR in two (4%) isolates. Earlier only one MDR strain of *S. pneumoniae* had been reported so far from India.[4] Increase in the number of MDR strains is a matter of worry as it will increase the economic and health burden for the patient.

An E-test has been seen to be a good alternative for testing MIC for *S. pneumoniae*.[19] It is a novel innovation, based on the principle of both the diffusion and dilution methods. Hence, this method was used in the present study, to find out the MIC for all other drugs except penicillin. Maximum resistance for cotrimoxazole and minimum for cefotaxime, as indicated by the E-test, correspond with other studies.[4] High resistance (14%) observed for ciprofloxacin in the present study can be attributed to more usage of quinolones these days. However, earlier studies have also mentioned the increasing trend for quinolone resistance in *S. pneumoniae*.[28–30] The resistance pattern for cefotaxime has shown a huge difference when using disk diffusion (30%) as against the E-test (0%). It can be explained on the basis that 30 μg cefotaxime disks (as used in the present study) and 30 μg ceftriaxone disks have not been entirely satisfactory because of an excessive number of minor interpretive discrepancies between disk tests and microdilution tests.[31] A 1 μg cefotaxime disk has been found to give better correlation with MIC categories, but such disks are not available commercially.[31] At present, neither cefotaxime nor ceftriaxone disks are recommended; rather, agar dilution, broth dilution, or E-test must be done. Some pneumococci with either intermediate or high-level penicillin resistance may also be resistant to extended-spectrum cephalosporins; although we have not isolated such strains, but it is always recommended that penicillin-resistant isolates be tested by MIC for susceptibility to either ceftriaxone or cefotaxime.[32]

During the late 1980s, the identification of a high prevalence of erythromycin-resistant strains in South Africa that was associated with multiple resistance in pneumococci (isolated from healthy children in the community) led to the concern that resistance may increase in countries where the drug was widely used.[33–35] In our set up, resistance to erythromycin was observed in 14% of the cases. Macrolide resistance in case of *S. pneumoniae* was due to either of the two mechanisms – modification of the drug binding site regulated by the erm(B) gene, or due to the active efflux mechanism, which is regulated by mef(A) gene.[36] The former mechanism led to high-level resistance (MIC > 64 mg/L), whereas, the latter led to low-level resistance (MIC 1-32 mg/L). In the present study the MIC of erythromycin was in the range of 0.001-5 mg/L. Therefore, most probably the resistance was due to mef (A) gene, which could be confirmed by the molecular method only.

### Strength of the study

This study suggests the possible emergence of penicillin-resistant as well as multidrug-resistant *S. pneumoniae* strains in the community. Penicillin as the best empirical choice for treatment of infections with *S. pneumoniae* may need reconsideration. It can no longer be considered a drug of choice in the treatment of life-threatening invasive conditions caused by *S. pneumoniae*. Our study also pointed out that not only penicillin, but other alternative antibiotics such as erythromycin, ciprofloxacin, and cotrimoxazole showed resistance against the isolates, and should be used carefully in future. This study will help the clinicians as well as microbiologists to treat the patients better in case of infection with *S. pneumoniae*.

### Limitations and future directions of the study

This study was planned as a pilot, so the number of clinical isolates was relatively small. It may not reflect the national status of pathogen distribution or the antimicrobial resistance pattern. This study will further continue to document the predominant serotype and the nature of genetic diversity in this region, to be further compared with the national scenario.

## CONCLUSIONS

Indiscriminate use of antibiotics at an inappropriate dosage at the community level might be the probable cause of resistance. Therefore, there should be a restraint for the indiscrete use of antibiotics, to limit the surfacing of resistant strains. Emergence of resistant strains and also the MDR strains of *S. pneumoniae* need continuous local as well as global monitoring of the sensitivity pattern, so as to plan the line of treatment.
